# Investigations Concerning Texas or Southern Cattle-Fever

**Published:** 1895-07

**Authors:** N. C. Powell, F. A. Hamilton

**Affiliations:** Senior Student of Veterinary Medicine, Ohio State University, Columbus, O.; Senior Student of Veterinary Medicine, Ohio State University, Columbus, O.


### INVESTIGATIONS CONCERNING TEXAS OR SOUTHERN CATTLE-FEVER.

BY N. C. POWELL AND F. A. HAMILTON,
SENIOR STUDENTS OF VETERINARY MEDICINE, OHIO STATE UNIVERSITY, COLUMBUS, O.

For the history of this disease and an account of its peculiarities, its symptoms, and the characteristic morbid changes occurring in individual animals, we refer all those interested to the abundant and accessible literature upon the subject.

In our work we will confine ourselves to a discussion of the various theories advanced to account for the phenomena observed in the spreading of this disease, and relate our experiments, made for the purpose of testing the tick (*ixodes bovis*) theory of communication.

It has been observed that this disease follows the course of the transportation of Southern cattle to Northern markets. These cattle, while themselves perfectly well, carry with them some contagium or infectious principle, which, when coming in contact with native Northern cattle, produces in the latter the pathological conditions which are designated "Texas or Southern cattle-fever." Thus it has been observed that Northern cattle, when transported in cars in which Southern cattle have been shipped, when occupying yards in which Southern cattle have been kept, when pastured or allowed to eat where Southern cattle have been grazing, or allowed to drink where Southern cattle have been watered, almost invariably contract the disease. It, therefore, appears that we have in this disease exactly the opposite conditions which exist in other infectious diseases, in that the disease is spread by perfectly healthy animals, while animals suffering from an attack are powerless to communicate it to other animals.

Perhaps the best explanation of these (paradoxical) facts is offered by the parasite theory, *i. e.*, by assuming that the disease is produced by (parasitic) micro-organisms which either adhere externally to the bodies of the Southern cattle or exist and are conveyed within their organism.

Various efforts have been made to discover the true micro-organism, fulfilling in every respect the conditions necessary to constitute the cause of this peculiar disease. Of these, we may mention the efforts of Dr. H. J. Detmers, of Columbus, O., to find the suspected germ in artificial cultures of the bacteria occurring in infusions of the native Texas grasses, and in the blood and tissues of cattle dying of this disease. He was unable to arrive at a definite conclusion.

Since then other investigators have boldly announced that they have found the specific micro-organism, the true cause of the disease. But a careful analysis of these statements shows them to be insufficiently supported by actual facts.

Notwithstanding these negative results, it may be considered very probable that the contagium or infectious principle is to be found adhering to the external surface of the bodies of the animals, or exists in the excretions, or, as supposed by Dr. Detmers, in the saliva or slaver of the Texas cattle.

Another theory worthy of consideration, advanced by some cattlemen as early as the seventies, and recently resurrected by Dr. D. E. Salmon and others, is that the ticks (*ixodes bovis*) which infest Southern cattle are the medium through which the disease is communicated. Along this line Salmon has made experiments. He put Northern cattle in fields which had been previously occupied by Southern cattle, and produced the disease in almost every instance. He also put Northern cattle in fields previously occupied by tick-free (?) Southern cattle, and failed to produce the disease. Again, he prepared fields by scattering over them ticks imported from the Southern States, and when Northern cattle were kept confined in these fields, Texas fever appeared among them. Furthermore, he claims to have produced unmistakable Texas fever in Northern cattle by applying to them young ticks artificially hatched in the laboratory. Along this latter line of investigation we have conducted our experiments. In our work we have been greatly aided by the kind assistance of Dr. M. Francis, Professor of Veterinary Science in the A. and M. College of Texas. To him we are indebted for our knowledge of the natural history of these parasites, and for our supplies of live ticks.

On March 24, 1895, the first consignment of ticks was sent, and it arrived in Columbus, safe and sound, on the 26th. This shipment consisted, at a moderate estimate, of 200 mature ticks, mostly females. These were placed in a glass dish in the incubator of the bacteriological laboratory, and kept at a temperature of 27° C. On April 3d we received the second shipment of live ticks from Texas. These, too, were placed in the incubator with the others. In a few days they began to deposit their eggs. By April 10th an immense number of eggs had been deposited, and the old ticks, with but few exceptions, were dead. The old ticks were then removed and destroyed, and the eggs kept in the incubator as before. Care was taken to keep the air in the incubator moist by keeping in it a vessel filled with water. By the 27th of April some of the eggs were noticed to be turning a sort of silver white, and on the 3d of May the first young ticks appeared. Only a few of the eggs of the first lot of ticks hatched, but from the eggs of the second lot we received quite a large number of ticks. On the 6th of May these young ticks were placed on a cow, a grade Jersey, probably about six years old, in good condition as to flesh, apparently healthy, except incipient tuberculosis, and had never, in any way been exposed to Texas fever. The ticks were placed on a cloth, and the cloth was tied against the abdomen immediately in front and over the udder. The young ticks soon attached themselves to the hair, and the cloth was left in position for twenty-four hours. As direct sunlight is known to be fatal to young ticks, the cow was kept in the stable the entire time of the experiment.

The rectal temperature, taken at the time the ticks were placed on the cow and twice daily afterward, was as follows:

| | A.M. | P.M. | | A.M. | P.M. |
|---|---|---|---|---|---|
| May 6th . . | 99.2° | 102.4° | May 19th . . | 101° | 101.6° |
| " 7th . . | 99.2 | 101.4 | " 20th . . | 100.2 | 101.2 |
| " 8th . . | 99.2 | 101 | " 21st . . | 100.2 | 101.6 |
| " 9th . . | 100 | 101 | " 22d . . | 100.6 | 101.8 |
| " 10th . . | 101 | 101 | " 23d . . | 100.4 | 101.8 |
| " 11th . . | 101.2 | 100.8 | " 24th . . | 101 | 102 |
| " 12th . . | 99.8 | 101.4 | " 25th . . | 100.8 | 102.4 |
| " 13th . . | 101.2 | 100.8 | " 26th . . | 100.6 | 101.2 |
| " 14th . . | 99.8 | 99.8 | " 27th . . | 100.6 | 101.6 |
| " 15th . . | 100.8 | 101.6 | " 28th . . | 102 | 101.6 |
| " 16th . . | 100 | 101.4 | " 29th . . | 101 | 102.4 |
| " 17th . . | 101.4 | 101.8 | " 30th . . | 101.4 | 100.8 |
| " 18th . . | 100.8 | 101.8 | " 31st . . | 100.6 | 101.6 |

On the 16th of May we received a third lot of ticks from Texas; but as no more cattle were available for experimental purposes, and as there was not any more sufficient time for anything to develop before the close of the term, we could not make any use of them.

However, we made artificial cultures of the micro-organisms found in and upon the ticks.

1. By puncturing the body of the tick with a sterilized platinum needle, and inoculating nutrient gelatin in a test-tube with the blood adhering to the needle. 2. By rubbing the sterilized needle on the body of the tick, and making puncture cultures with what adhered. 3. By putting an entire tick into a test-tube with nutrient gelatin.

In three to four days the cultures from the blood of the ticks were growing rapidly, the entire length of the puncture, but most at the surface, and liquefying the gelatin. Cover-glass preparations showed them to be pure cultures of a short bacillus, about the size of bacillus prodigiosus. They showed a strong tendency to form chains or threads. The puncture-cultures made from the external surface of the tick grew slowly along the entire length of the culture, but best at the surface, and did not liquefy the gelatin. Cover-glass preparations showed micro-organisms resembling ordinary cocci. The cultures produced by putting the entire tick in the gelatin-tube were composed of the same or similar cocci, but growing more rapidly, because more on the surface.

The young ticks, when put on the cow, May 6th, were scarcely as large as a pin's head, but in the course of a week or two they could be plainly felt beneath the hair. They grew rapidly, and toward the latter part of May they were almost full-grown, and could readily be seen adhering to the skin.

Our experiment, made on only one animal, of course, cannot have the value, and cannot be as decisive as it would have had, and been, if it had been more extensive and been made on a large number of animals, which, unfortunately, were not at our disposal. All that we claim is this: It is one instance in which no signs or symptoms of Texas fever were produced during and beyond the time specified by the advocates of the tick theory as the period of incubation, notwithstanding that all the necessary conditions to produce Texas fever, as laid down by the advocates of the tick theory, have been fully complied with. We will yet state that although the time at our disposal for our experiment expired June 1, the cow has been kept under strict observation until June 12, and no signs or symptoms of Texas fever have made their appearance.

